# Retraction: MyoD-dependent regulation of NF-κB activity couples cell-cycle withdrawal to myogenic differentiation

**DOI:** 10.1186/2044-5040-3-15

**Published:** 2013-07-18

**Authors:** Maura H Parker, Julia von Maltzahn, Nadine Bakkar, Ban Al-Joubori, Jeff Ishibashi, Denis Guttridge, Michael A Rudnicki

**Affiliations:** 1 Faculty of Health Sciences Graduate Programme, McMaster University, Hamilton, ON, Canada; 2 Ottawa Hospital Research Institute, Molecular Medicine Program, Ottawa, ON, Canada; 3 Current address: Fred Hutchinson Cancer Research Center, Clinical Research Division, Seattle, WA, USA; 4 The Ohio State University College of Medicine, Columbus, OH, USA; 5 Ottawa Hospital Research Institute, 501 Smyth Rd, Ottawa, ON K1H 8L6, Canada; 6 Greg W Fulton ALS and Neuromuscular Research Center, Barrow Neurological Institute, Phoenix, AZ, USA

## Retraction notice

The authors would like to retract the article "MyoD-dependent regulation of NF-κB activity couples cell-cycle withdrawal to myogenic differentiation" [1]. After the article was published, the first author revealed that the tubulin blots in Figures 1A, 3E, and 4A are from unrelated samples. The lead author and co-authors apologise to the readers, reviewers and editors for publishing this erroneous data.
